# Preformed expression of defense is a hallmark of partial resistance to rice blast fungal pathogen *Magnaporthe oryzae*

**DOI:** 10.1186/1471-2229-10-206

**Published:** 2010-09-17

**Authors:** Emilie Vergne, Xavier Grand, Elsa Ballini, Véronique Chalvon, P Saindrenan, D Tharreau, J-L Nottéghem, J-B Morel

**Affiliations:** 1 INRA, UMR BGPI INRA/CIRAD/SupAgro, Campus International de Baillarguet, TA A 54/K, 34398 Montpellier, France; 2 Montpellier SUPAGRO, UMR BGPI INRA/CIRAD/SupAgro, Campus International de Baillarguet, TA A 54/K, 34398 Montpellier, France; 3 CNRS-Université Paris-Sud, Institut de Biotechnologie des Plantes, Physiopathologie Moléculaire Végétale, Bâtiment 630, 91405 Orsay Cedex, France; 4 CIRAD, UMR BGPI INRA/CIRAD/SupAgro, Campus International de Baillarguet, TA A 54/K, 34398 Montpellier, France

## Abstract

**Background:**

Partial resistance to plant pathogens is extensively used in breeding programs since it could contribute to resistance durability. Partial resistance often builds up during plant development and confers quantitative and usually broad-spectrum resistance. However, very little is known on the mechanisms underlying partial resistance. Partial resistance is often explained by poorly effective induction of plant defense systems. By exploring rice natural diversity, we asked whether expression of defense systems before infection could explain partial resistance towards the major fungal pathogen *Magnaporthe oryzae*. The constitutive expression of 21 defense-related genes belonging to the defense system was monitored in 23 randomly sampled rice cultivars for which partial resistance was measured.

**Results:**

We identified a strong correlation between the expression of defense-related genes before infection and partial resistance. Only a weak correlation was found between the induction of defense genes and partial resistance. Increasing constitutive expression of defense-related genes also correlated with the establishment of partial resistance during plant development. Some rice genetic sub-groups displayed a particular pattern of constitutive expression, suggesting a strong natural polymorphism for constitutive expression of defense. Constitutive levels of hormones like salicylic acid and ethylene cannot explain constitutive expression of defense. We could identify an area of the genome that contributes to explain both preformed defense and partial resistance.

**Conclusion:**

These results indicate that constitutive expression of defense-related genes is likely responsible for a large part of partial resistance in rice. The finding of this preformed defense system should help guide future breeding programs and open the possibility to identify the molecular mechanisms behind partial resistance.

## Background

Plants are constantly exposed to microbial attacks and have developed sophisticated systems to counteract them. Plants respond to infection using a two-layers innate immune system [1]: a first layer, basal resistance, responds to pathogen-associated molecular patterns (PAMPs). Basal resistance is though to be the default defense system that allows limited restriction of pathogen growth. A second layer, gene-for-gene resistance, responds to pathogen virulence factors. Both basal and the gene-for-gene induced resistances can generally be divided into three steps. In a first step, the plant throughout different recognition systems detects PAMP or virulence effectors of the pathogen; these recognition systems involve pattern recognition receptors (*PRRs*) for basal resistance and resistance (*R*) genes for gene-for-gene resistance [1,2]. In rice, the transmembrane glycoprotein CEBiP is the best-characterized example of PRR for basal resistance to the fungal pathogen *Magnaporthe oryzae *[3]. There is little polymorphism in the case of *PRR *and in the molecular pattern that they recognize. The gene-for-gene recognition system is much more polymorphic. Depending on the presence/absence of the *R *genes and of the corresponding pathogen molecule, the interaction will be incompatible (plant is resistant) or compatible (plant is susceptible).

In a second step, signal transduction occurs and requires regulators such as MAP kinases [4] and transcription factors [5]. These genes that are here collectively called defense regulators are often conserved across species; for example *NPR1 *is a central regulator in both Monocots and Dicots [6-10]. Many of these regulator genes are differentially expressed during infection [11,12].

In a third step, defense responses are induced. These include production of antimicrobial secondary metabolites (phytoalexins) [13], pathogenesis-related (PR) proteins (e.g. chitinases, glucanases) [14,15], cell-wall strengthening [16] and programmed cell death, leading to the Hypersensitive Response (HR) [17]. The genes that act downstream of the regulators controlling the disease resistance pathways are collectively called defense genes and are typically transcriptionally regulated upon infection.

Besides these mechanisms explaining how resistance is built, breeders and biologists use an agnostic but operational term for a phenomenon found in many plant species: partial resistance. Partial resistance is first characterized by quantitative limitation of pathogen growth. In rice, partial resistance to the blast fungus *M. oryzae *is often divided into two main values: the number and the size of lesions [18]. Another characteristic of partial resistance is that it is controlled by the plant development and usually increases with aging [19]. Rice is a good model to study partial resistance as breeders have extensively used it, through the identification of quantitative trait loci (QTL). There is a considerable amount of genetic data available that was recently reviewed [18]. More than 340 QTL have been identified and summarized to 165 metaQTLs. Further analysis lead to the identification of an operational set of about 20 genomic areas. Importantly, this large set of genetic data could be compared to the large set of information available on *R *gene analogs, regulators and defense genes in rice [12,18]. This analysis showed that, on a global scale, *R *gene analogs are often found in intervals defining metaQTLs [18]. This was an expected finding consistent with the hypothesis that partial resistance is due, in part, to defective *R *genes that recognize with low efficiency pathogens and trigger weak defense response. Less expected was the finding that regulator and some defense genes were also significantly associated with metaQTLs [12]. Finally, partial resistance has long been considered as a durable form of resistance. This may be due to the fact that the low levels of resistance conferred by partial resistance do not impose strong selection pressure for the pathogens. This may also be due to particular mechanisms that cannot be easily broken down by pathogens.

Preformed, constitutive, physical and chemical barriers likely play a role in partial resistance by limiting the growth of a normally virulent pathogen. They involve cuticle [20] and cell wall strengthening [21] and represent mostly broad-spectrum pathogen resistance. In rice, like in other plants, silicon accumulation plays a direct role in partial resistance by activating some defense mechanisms [22] and an indirect role by deposition beneath the cuticle to form a cuticle-silicon double layer which can mechanically impede penetration by fungi and thereby disrupt the infection process [62]. Antimicrobial molecules, called phytoanticipins, can also accumulate before infection [23]. Although there is a large body of evidence that defense genes, especially pathogenesis-related (PR) proteins, are constitutively expressed in uninfected tissues [15], there is no indication of the effect of their level of expression before infection on resistance. In contrast, there are many indications that the over-production of PR proteins confers resistance [24,25], that mutations in genes negatively regulating disease resistance can increase defense gene expression [e.g. [26,27]] or that over-expression of regulator genes acting positively on disease resistance can increase defense gene expression [e.g. [28]]. Thus there are indirect evidences that constitutive expression of regulator and defense genes could participate to plant pathogen resistance.

To face pathogen attacks, plants could use a proactive strategy of constitutive expression of inducible defense systems. Recently, large-scale expression studies across *Arabidopsis thaliana *cultivars have been completed and showed that gene expression greatly vary from one genotype to another [29]. Interestingly, the 2,200 differentially expressed genes were significantly enriched for genes classified as controlling biotic and abiotic responses [29]. Thus these classes of genes seem to display high expression level polymorphism (ELP). However, there is little information of a possible link between these ELPs and biological traits. ELP of major *R *genes can obviously explain the polymorphism in the disease resistance pathway [30,31]. In these cases, the presence/absence of the resistant *R *allele explains the ELP and the corresponding resistance/susceptibility phenotypes. In the case of partial resistance, there is no evidence that plants show ELPs of the surveillance receptors and/or regulator and defense systems. Our hypothesis is that such expression level polymorphism for receptors, regulator and defense genes belonging to plant disease resistance pathways play a role in partial resistance.

In this study, we wanted to test the hypothesis that, besides inducible defense systems, rice has developed a proactive strategy to face its major fungal pathogen, *M. oryzae*. For this purpose, we looked for possible links between constitutive levels of expression of genes markers of the disease resistance pathways (thereafter called defense-related genes) in relation to partial resistance. We show that constitutive expression of defense-related genes shows high ELP and likely plays a central role in partial resistance to *M. oryzae*. Thus we identify a possible mechanism underlying a phenomenon that has been known and used for a long time with no comprehensive knowledge of what was behind.

## Results

### Sampling rice diversity for partial resistance

**Figure 1:**
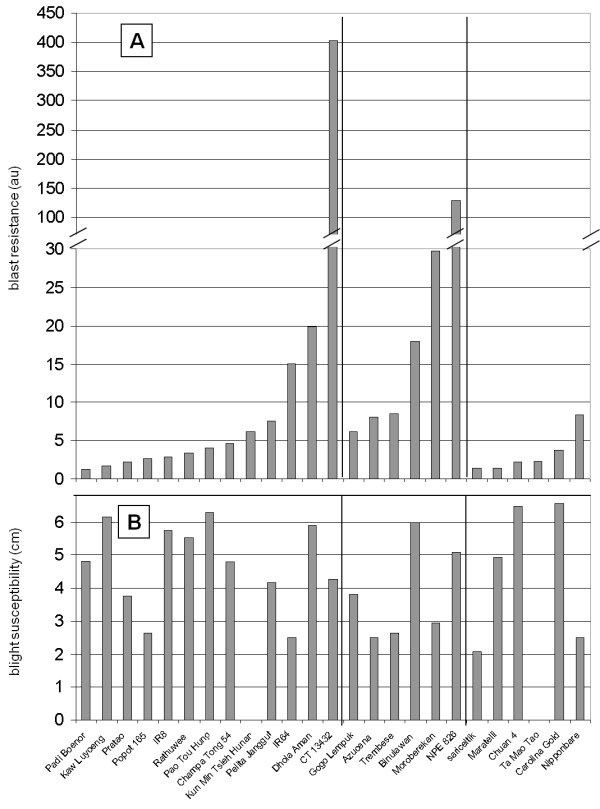
**Partial resistance to blast and bacterial blight**. (A) Partial resistance to blast fungus (*Magnaporthe oryzae*) was calculated according to fungal mass as measured by Q-PCR (see Methods and additional file 2). Three multivirulent isolates were used. The genotypes are displayed from the most susceptible to the most resistant. (B) Partial resistance to bacterial blight (*Xanthomonas oryzae *pv *oryzae *PX099) was estimated 15 days after inoculation and quantified by measuring the length of chlorosis from the inoculation section. The different rice sub-groups are separated by vertical bars (indica, tropical japonica and temperate japonica cultivars from left to right)

The goal of this study was to try to establish possible links between the constitutive expression of defense-related genes and partial resistance. Our approach was first to evaluate rice diversity (indica and japonica sub-groups) for partial resistance, trying to avoid resistance phenotypes resulting from gene-for-gene interactions. The analysis of partial resistance thus requires the removal of necrotic, HR-like, lesions that could result from defeated *R *genes triggering attenuated gene-for-gene resistance [32]. To meet this criterion, we selected rice/*M. oryzae *interactions that were as close as possible to compatibility. Based on preliminary results (JL Nottéghem, personal communication), we selected 23 rice cultivars. We also included five rice accessions that are commonly used in the research community and for which genomic and/or genetic information exists (IR64, Nipponbare, Azucena, Maratelli and Sariceltik). These cultivars represent 57% of overall rice allelic diversity, 51% for japonica sub-group and 55% for the indica sub-group as estimated by allelic diversity of microsatellites markers (Garris et al, 2005; Additional file 1). These 28 rice accessions were inoculated with four multivirulent isolates with broad-spectrum virulence (see Methods and Additional file 2), and partial resistance was estimated. An index was created for partial resistance that measures fungal growth *in planta *as quantified by Q-PCR (see Methods and Additional files 1 and 3). In calculating the partial resistance index, we were careful to remove as much as possible background gene-for-gene interactions. These were manifested by extremely low quantities of fungal growth and/or HR-like lesions. Out of 25 rice accessions tested, 23 were finally selected as representing most of partial resistance quantitative diversity. The genotypes showing high resistance against all *M. oryzae *strains (CT13432 and NPE826; Figure 1 and Additional File 2) were removed from the analysis as they likely reflect complete resistance driven by major *R-*genes. The well-known susceptible japonica genotypes Sariceltik and Maratelli were also found susceptible in this analysis. The indica genotype Padi-Boenor occurred to be the most susceptible in this assay. Thus, a total of 23 rice accessions were characterized for partial resistance to rice blast disease and bacterial blight (Figure 1).

From this analysis, it appears that rice accessions from the tropical japonica sub-group were over-represented among accessions with elevated levels of partial resistance to rice blast (Figure 1 and Additional file 3; 5/10 accessions). Partial resistance index ranged from 30 (tropical japonica Moroberekan) to 1.3 (indica Padi Boenor). Thus partial resistance to blast fungus is highly variable across rice diversity and can vary up to 23-fold. There was no obvious correlation between partial resistance to blast and bacterial blight.

### Constitutive expression of defense as a better indicator of partial resistance than inducible expression

We first address the question of the relative roles of inducible and constitutive expression of selected defense-related genes in partial resistance. For this purpose, we designed an experiment with a limited number of marker genes (11) and six representative rice accessions. This experiment was repeated three times independently to monitor gene expression before infection and 1, 2, 3 and 4 days post-inoculation (dpi).

**Figure 2:**
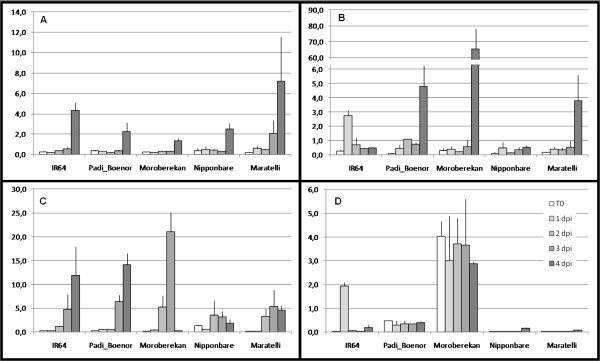
**Variability of defense induction across rice diversity**. Expression given in arbitrary unit (au) was measured by QRT-PCR before inoculation (T0) and 1, 2, 3 and 4 day(s) post inoculation (dpi) with the CD203 *M. oryzae *isolate in two or three biological repetitions. The expression of four genes is shown: *POX223 *(A), *RBBI2 *(B), *PBZ1 *(C) and *BURP *(D). Moroberekan is a tropical japonica cultivar that shows strong partial resistance. Nipponbare and Maratelli are temperate japonica cultivars that show respectively strong and weak partial resistance and IR64 and Padi Boenor are indica cultivars that show respectively strong and weak partial resistance

**Figure 3:**
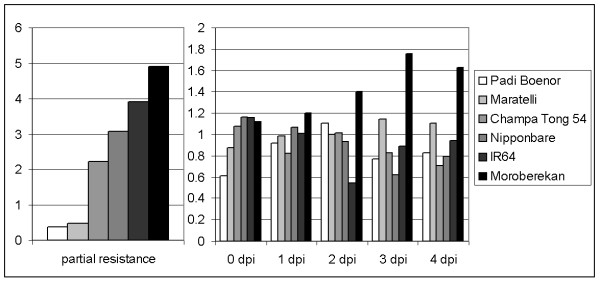
**Constitutive and inducible expression of defense genes**. The expression of the 21 genes (Additional File 4) was measured before and after inoculation with the *M. oryzae *isolate CD203 and compared to partial resistance index (A) as measured in Figure 1. A gene expression index (B), integrating all 21 gene expression values for each condition, was calculated as indicated in Additional file 5. Partial resistance and gene expression indexes correlate before infection (0 dpi) but not after (1 to 4 dpi)

This experiment indicated that most of the defense-related genes selected were induced after infection (Figure 2 and Additional file 4). In order to compare partial resistance to gene expression, we built an expression index that takes into account the expression values of all genes (See Methods and Additional file 5). We could then compare the partial resistance index to the expression indexes at the different times before and after infection (Figure 3).

Regression analysis (Additional file 6) suggests that there is a good correlation between both indexes before infection (R^2 ^= 0.8, p < 0.0027) but no statistically significant correlation of these indexes after infection. Thus the data on expression of this selected marker genes evaluated on this small set of rice cultivars suggests that expression before infection, more than after infection, correlates with partial resistance.

### The level of constitutive expression of defense-related genes is highly polymorphic across rice diversity

We wanted to further extend this analysis to a larger set of rice genes and accessions. We measured constitutive gene expression of 21 genes representative of the rice defense arsenal (Additional file 4) in the 23 rice accessions for which we measured partial resistance (Additional file 3). Constitutive expression was measured in seven independent experiments at the time when inoculation is usually performed and when partial resistance has started to develop (3 weeks after sowing).

When treated individually, the constitutive expression of each gene revealed several points (Additional file 7). First, we observed an important variability of the expression levels across cultivars. For example, the expression level of the classical defense gene *PBZ1 *vary up to 35-fold (Additional file 7), with a value of 0.02 in one of the most susceptible cultivar, Sariceltik, and a value of 0.7 in the most resistant cultivar, Moroberekan (Additional file 3). Second, the pattern of constitutive expression was sometimes different between the indica and japonica rice sub-groups (e.g. the *BURP *gene, Additional file 7).

**Figure 4:**
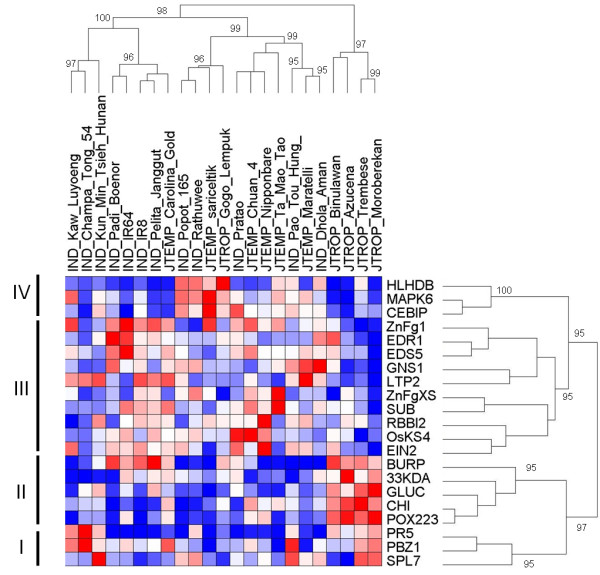
**Constitutive expression of defense systems across rice diversity**. The gene expression values before infection of 21 genes of 23 rice cultivars (IND = indica, JTROP = tropical japonica, JTEMP = temperate japonica) were used for hierarchical clustering using GenePattern analysis platform (http://www.broadinstitute.org/cancer/software/genepattern/index.html). Pearson correlation was used as distance and a pairwise complete-linkage as clustering method for both genes and varieties. Bootstrap values were estimated on 1000 permutations by the approximately unbiased method using R package pvclust [57]. Only boostrap values above 95 are shown. Each point represents the mean of three independent experiments. Most of the tropical japonica cultivars show a distinct expression pattern as compared to the other cultivars. The regulons are indicated by roman numbers

We used hierarchical clustering to identify groups of genes that were co-regulated across rice diversity (Figure 4). Several groups of genes that are co-regulated were found, as supported by bootstrap analysis. The first group (regulon I) contains both *PR *genes and regulatory genes (*PBZ1*, *PR5 *and *SPL7*). The second group (regulon II) mostly contains *PR *genes (*BURP*, *33 kDa*, *GLUC*, *POX223 *and *CHI*). The third group (regulon III) consists in a last large group of genes that contains both regulatory and *PR *genes. The last group (regulon IV) contains genes involved in recognition (*CEBiP*) and signal transduction (*MAPK6*, *HLHDB*).

Hierarchical clustering of the data also confirmed that the genetic background considerably affects constitutive expression of the selected genes (Figure 4). For instance, the sub-group of tropical japonica rice appeared clearly different from the other genetic groups of rice. This difference is mostly due to genes from regulons I and II. Thus, rice cultivars from different sub-groups display contrasting capacities to express defense-related genes before infection, suggesting contrasting regulation capacities.

### The level of constitutive expression of defense-related genes strongly correlates with partial resistance across rice diversity

**Figure 5:**
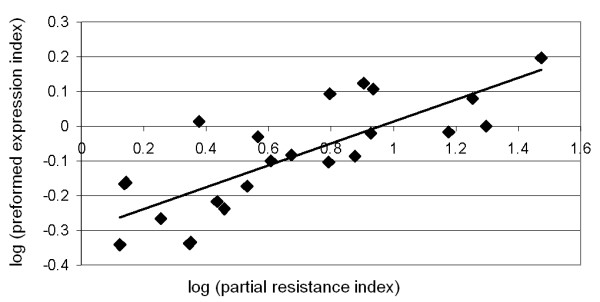
**Partial resistance and constitutive expression of defense correlate**. The log value of partial resistance (X-axis; Additional file 3) and expression of preformed expression of 21 genes (Y-axis; Additional File 4) indexes of the 23 representative rice cultivars was plotted. Correlation coefficients were statistically tested using the Pearsons' product moment correlation coefficent test and the Bonferroni correction (the initial 0.01 threshold was divided by 3 because each data set was tested 3 times)

It was noteworthy from previous observations that the tropical japonica subgroup is also the genetic sub-group displaying the highest partial resistance index in our analyses (Figure 1 and Additional file 3). In order to search for global correlations between constitutive expression of tested genes and the measured partial resistance index, the expression data of the 21 selected defense-related genes in the 23 rice genotypes was analyzed using the expression index already used (See Methods and Additional file 5). We found a strong correlation between constitutive expression of defense-related genes and partial resistance (R^2 ^= 0.83, p < 1.756e-6; Figure 5). Thus, the previous observation on a small subset of rice diversity (Figure 3 and Additional File 6) holds true when tested on a sample of cultivars representing a large subset of rice diversity. Similar results were also found when separately testing indica and japonica sub-groups (Additional file 8).

Using Principal Component Analysis (Additional file 9) and ANOVA (Additional file 10), we could identify the genes that, in our selection, were the most significantly reflecting the correlation between constitutive expression of defense and partial resistance. The *PBZ1 *gene from regulon I was found to be the best marker of constitutive expression of defense-related genes across indica and japonica rice sub-groups. Thus, for the *PBZ1 *gene, the observed correlation between constitutive expression and partial resistance holds true for almost all the 23 rice genotypes tested, despite the diversity explored. Another gene from regulon I, the *SPL7 *gene, appeared to be a good marker for indica genotypes. The *BURP *and *GLUC *genes from regulon II were good markers for the japonica sub-group. Overall, this analysis across rice diversity suggests that constitutive expression of defense-related genes and partial resistance are highly correlated.

### Constitutive expression of defense-related genes is developmentally controlled

**Figure 6:**
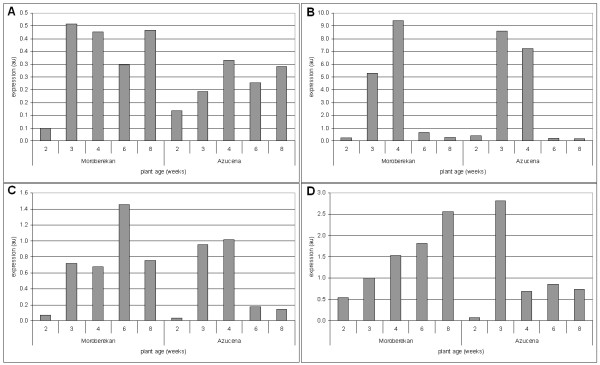
**Developmental control of preformed expression of defense in tropical japonica rice**. The last leaves of plants at different developmental stages (2 to 8 weeks) were simultaneously harvested and analyzed for preformed expression of defense. Two tropical japonica cultivars (Moroberekan and Azucena) were selected as representative of cultivars showing high preformed expression of defense. The example of four genes is shown: *POX223 *(A), *RBBI2 *(B), *PBZ1 *(C), *SPL7 *(D) and similar results were found with four other genes (data not shown)

Partial resistance is well known to increase along plant development [19]. In particular, in rice there is a strong difference between resistance to blast in a 2-weeks old plant (juvenile-susceptible) and resistance in a 3-week old plant (young adult-resistant). It is also quite common that the last emerged leaf (leaf n) is often more susceptible than the leaf that emerged one week before (leaf n-1). We thus tested whether constitutive expression of defense-related genes was following the same developmental patterns. We chose the tropical japonica cultivars Moroberekan and Azucena, as they are good representative of constitutive expression of defense-related genes (Figure 4 and Additional file 3). Constitutive expression of defense-related genes was measured in plants aged from 2 to 8 weeks, on two different leaves (last and before the last leaf emerged). As shown in figure 6, the expression of these defense-related genes followed the same developmental pattern than partial resistance with a strong increase in expression between two and three weeks after sowing. This was true for the eight marker genes tested (data not shown). This increase of expression was maintained for half of the genes tested. We have yet no explanation for the decrease of expression of some genes like *RBBI2 *later in development. We also observed that constitutive expression of defense-related genes was overall higher in leaf n-1 than in leaf n (Additional file 11), a pattern very similar to age-related partial resistance. Thus, this striking parallel between partial resistance and expression of defense-related genes during plant development further supports our hypothesis that partial resistance can be explained by constitutive expression of defense-related genes.

### Constitutive level of SA and ethylene do not explain partial resistance

There is a previous report that salicylic acid (SA) a signaling molecule involved in disease resistance could play a role in partial resistance of rice to *M. oryzae *[33]. Jasmonic acid (JA) [34] and ethylene [35] are also identified as important signaling molecules in plant disease resistance. We asked whether these signaling molecules could relate to constitutive expression of defense-related genes. We evaluated the SA and ethylene pathways by direct quantification of total SA and ethylene in 3-week old plants. We monitored the implication of the JA pathway by using the marker gene *RCI1 *[36].

The constitutive expression of *RCI1 *did not correlate with partial resistance to *M. oryzae *(Additional files 9 and 10). Thus the JA constitutive levels, as monitored by the *RCI1 *gene, do not seem to contribute to partial resistance.

**Figure 7:**
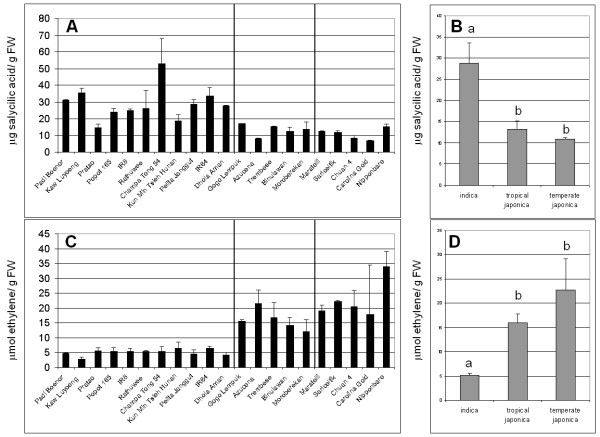
**Preformed quantities of salycilic acid and ethylene in rice cultivars**. Constitutive amounts of salycilic acid (A) and ethylene (C) were measured in the absence of infection. Each point represents the mean and standard deviation of two separate assays. The vertical lines separate, from left to right, indica, tropical japonica and temperate japonica genotypes. For each sub-group, the cultivars are displayed from the less to the more resistant. The average values in the different rice sub-groups are also shown for salicylic acid (B) and ethylene (D). The letters (a or b) above the bars indicate whether the average value of salicylic acid levels (B) or ethylene levels (D) are significantly different between each sub-groups as evaluated by a Student tests (P < 0.005)

Total SA and ethylene were directly extracted and quantified. The amount of these two molecules was very different across rice diversity (Figure 7). In each rice sub-group, the constitutive quantities of SA or ethylene were similar in rice accessions showing elevated and weak partial resistance (Figure 7A and 7C). We could not detect any correlation between the level of partial resistance and the levels of SA or ethylene (data not shown). However, we observed that constitutive amounts of SA are 2-fold higher in indica cultivars than in japonica cultivars and this difference is statistically significant (Figure 7B). Conversely, the constitutive levels of ethylene were higher in japonica than in indica cultivars (Figure 7D). Thus, SA and ethylene constitutive levels negatively correlate (R^2 ^= -0.81, P < 5.2 × 10^-6^). Although we detected a high level of polymorphism for SA and ethylene in rice, we could not find any correlation between these molecules and partial resistance, nor with constitutive expression of defense-related genes.

### Co-localization of QTLs controlling constitutive expression of defense-related genes and QTLs for partial resistance

A prediction of our hypothesis is that we should be able to find areas of the rice genome that control both constitutive expression of defense-related genes and partial resistance. In order to identify such regions, we initiated a QTL analysis on gene expression. Expression data has been recently used as quantitative traits for QTL analysis [37]. The resulting QTLs are called eQTLs, for expression QTLs. Two types of eQTLs are expected: cis-eQTLs that are located at the same locus that the gene monitored for expression (structural gene) and trans-eQTLs that are located at another locus.

We used two japonica X indica mapping populations: the Moroberekan X CO39 population with 60 recombinant inbred lines (RILs) [38] and the Azucena X IR64 population with 84 RILs [39]. Among the genes tested in this study (Additional File 4), we looked for genes that would show the strongest constitutive expression polymorphism between the parents of the available RIL populations (data not shown). The *BURP *and *CHI *genes showed the strongest polymorphism and were chosen for eQTL analysis. For each mapping population, two to three independent experiments were done in which constitutive expression of these genes was monitored as well as disease symptoms and used as quantitative traits.

**Figure 8:**
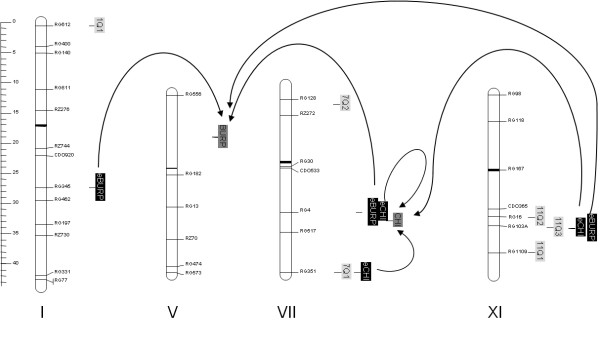
**Simplified QTL and eQTL maps for blast disease resistance and constitutive expression**. QTLs (towards CD203 isolate) are indicated in light grey squares, eQTLs (for the structural genes *BURP *and *CHI*) in black boxes and structural genes in dark grey boxes. The arrows indicate the positive effects of eQTLs on structural genes. Genetic markers used for QTL analysis are indicated on the right of each chromosome. Only chromosomes showing significant eQTLs (LOD score > 3) are shown. This map was obtained using the Moroberekan X Co39 RILs population; similar results were obtained using the IR64 X Azucena population (data not shown).

The Figure 8 shows the eQTL and QTL detected with LOD > 3 (Additional File 12) in at least two independent experiments (false discovery rate of 0.001). Three eQTLs (chromosome 1, 7 and 11) for the *BURP *gene and three eQTLs for the *CHI *gene (two on chromosome 7 and one on chromosome 11) were found. Most of them were trans eQTLs. One cis-eQTL was detected for the *CHI *gene. Quite remarkably, two eQTLs were common to the *CHI *and the *BURP *genes, suggesting that the constitutive expression of these genes could be controlled by the same locus. The eQTLs for *BURP *and *CHI *found on chromosomes 1 and 7 respectively were observed in both mapping populations, further supporting the existence of eQTLs in these regions. In all cases the favorable allele increasing constitutive expression was from the tropical japonica parental line (Moroberekan and Azucena). For the *BURP *gene, this is consistent with the observed positive correlation between constitutive expression of this gene and partial resistance in japonica but not indica sub-group (Additional File 10).

Twelve QTLs for blast partial resistance were found (Additional File 12). It was striking that two of these QTLs, one on chromosome 7 (RG4 marker) and one on chromosome 11 (RG103A marker) are co-localizating with eQTL. This is the first genetic evidence that the control of constitutive expression of a defense-related genes could account for partial resistance.

## Discussion

### The expression of defense-related genes and molecules is highly polymorphic in rice

We decided to use rice diversity in order to establish a role of constitutive expression of defense-related genes in partial resistance. We analyzed 23 rice cultivars that were randomly selected in the two major groups of indica and japonica. These cultivars represent up to 57% of rice diversity and thus can be considered as a representative sample. In order to evaluate partial resistance for *Magnaporthe oryzae*, we generated an index that mostly takes into account fungal growth. Complete resistance driven by specific gene-for-gene interactions was removed. Overall, our quantitative index correlates with other measurements of partial resistance like lesion number (XG and JBM, data not shown).

Partial resistance to blast fungus did not correlate with quantitative resistance to rice blight (Figure 1). This may be due to different lifestyles of the fungal pathogen (hemibiotrophic, growing in the mesophyl) and bacterial pathogen (biotrophic, growing in the xylem) tested. This may also be due to the fact that partial resistance to blast was evaluated on 3-weeks old plants whereas resistance to bacterial blight was evaluated on 8-weeks old plants. Finally, resistance to blight may not involve the same components that resistance to blast.

The genes that were used to measure constitutive expression are representative of the disease resistance pathway. Most of the regulators have been demonstrated to be positive regulators of resistance by mutant analysis (See references in Additional File 4). Many of the defense genes studied have also been shown to increase resistance in plants that are over-expressing them [25]. Finally, the *OsKS4 *gene was selected as a representative gene for the momilactone biosynthetic pathway, one of the major rice phytoalexin [40].

It was clear from our analysis that constitutive expression of the defense-related genes selected was highly polymorphic across rice diversity (Figure 2 and Additional file 7). This analysis revealed that the tropical japonica sub-group displays a unique capacity to express defense-related genes before infection (Figure 4). This observation likely reflects that these genotypes possess polymorphic and/or unique regulators of disease resistance.

The constitutive amounts of signaling molecules like salicylic acid and ethylene is also extremely polymorphic (Figure 7), especially between indica and japonica cultivars. Comparing this polymorphism to expression polymorphism of other plant metabolic pathways would tell us whether these ELP (expression level polymorphism) are comparable or not. An analysis in Arabidopsis suggests that defense-related genes display elevated ELPs as compared to genes belonging to other pathways [29]. It is also noteworthy that sequence polymorphism of defense-related genes is low in Arabidopsis [42]. It is thus likely that disease resistance polymorphism results more from expression than from polymorphism at the protein level, with the exception of gene-for-gene polymorphism.

### Preformed defense, but not induced defense, is a hallmark of partial resistance to the rice blast fungus

Our hypothesis was that the constitutive expression of defense-related genes was contributing to partial resistance. This was initially based on observation on a few rice cultivars and genes (EV and JBM, data not shown). This was also motivated by several piece of literature reporting that physical barriers [20,22] and preformed antimicrobial molecules [23] play an important role in disease resistance. We thus addressed the question of a putative contribution of defense-related genes in general in partial resistance. We built an index of gene expression where all genes participate to the same extent to the final value (See Methods and Additional File 5). When compared to the index of partial resistance, we found a very strong correlation between constitutive expression of defense-related genes and partial resistance (Figure 5). This correlation was extremely robust as we observed it in seven independent experiments in the past two years. We conclude that this constitutive expression of defense-related genes is a preformed defense system that contributes to partial resistance.

The group of tropical japonica cultivars showed an atypical pattern of constitutive expression of defense-related genes (Figure 4). These cultivars also displayed a high index of partial resistance and of constitutive expression of defense. This observation suggests that this rice sub-group harbors a particular preformed defense system.

Some genes like the regulatory gene *SPL7 *and the *PR *gene *PBZ1 *were good markers of preformed defense (Additional Files 7 and 10). The only *PRR *gene tested here, the *CeBiP *gene, was not a good marker of preformed defense. Cloned *R *genes [18] and more recently identified receptor-like genes (*WAK1*) [43] need to be tested to determine if constitutive expression of defense-related genes involves all steps of the basal and gene-for-gene resistance pathways. Finding more genes with an expression pattern correlated to partial resistance will help us to build gene sets to further studying preformed defense.

There was no obvious correlation between partial resistance and expression after infection of the defense-related genes tested (Figure 3 and Additional File 6). Since the infection process of rice by *M. oryzae *occurs very early after inoculation, it is possible that we underscored early time points (less than 24 h after infection). It remains possible that gene expression in the very early steps of infection also correlate with partial resistance. It is also extremely difficult to estimate the part of partial resistance that can be attributed to preformed and induced defense. Identifying genes that control preformed but not induced defense could help defining the respective contribution of each system. Without rejecting the probable contribution of induced defense in partial resistance, our results strongly suggest that constitutive expression of defense-related genes highly contributes to partial resistance.

### Preformed defense parallels the developmental control of partial resistance

One of the best evidence for a contribution of preformed defense to partial resistance comes from the observation that these two phenomena are coordinated during development. Partial resistance is well known in rice to be developmentally regulated [19]. When we measured constitutive expression of defense-related genes, we observed that this expression was following the increase of partial resistance during plant growth. The constitutive expression of all genes tested dramatically increase between juvenile (2-week old plant) and young adult plants (3-week old plants) (Figure 6). Such a massive effect suggests that there is a major control of development on the expression of preformed defense. Recently, Zhao et al [45] also observed that the constitutive expression of the *R *genes *Xa3/Xa26 *and *Xa21 *were developmentally controlled. This could easily explain in this case why gene-for-gene resistance driven by these genes was effective in adult plants but not in juvenile plants. Finding common regulatory points between development and defense may help us understanding how partial resistance is developmentally controlled.

### The regulation of preformed defense has yet to be identified

In order to get further insights on the way preformed defense is deployed by rice, we tested the implication of three signaling pathways controlled by salicylic acid, jasmonic acid and ethylene in constitutive expression of defense-related genes. We did not find evidence that these pathways were involved. This was unexpected for the SA pathway since previous report [33] suggested that constitutive SA levels were correlated to *M. oryzae *resistance. When we closely examined this report, we found out that the disease index used by Silverman and colleagues was not defined and that disease resistance in their case most likely correlated with indica/japonica differences. This could simply reflect the fact that some *M. *oryzae isolates are better adapted on one rice sub-group than on another. We circumvented this difficulty by trying to incorporate in our disease index only partial resistance as monitored by using multivirulent isolates of *M. oryzae*. We conclude that neither constitutive expression of SA, JA nor ethylene pathway correlates with ELP of defense-related genes.

Alternatively, preformed defense could result from the leakage of the disease resistance pathways. For example, assuming that some signaling is constantly triggered by the environment, rice cultivars having efficient but leaky regulatory pathways would also display elevated levels of defense-related genes in the absence of infection. This mechanism would require positive regulators of disease resistance to be very active and negative regulators to be quite inactive. This mechanism would be similar to the mechanism by which the barley *mlo *gene confers resistance to powdery mildew. The *MLO *gene is a negative regulator of disease resistance and recessive alleles (*mlo*) of this gene confer broad-spectrum resistance [46]. Some *mlo *alleles are weak negative regulators such that the plant constitutively expresses parts of the disease resistance pathway, leading to spontaneous cell-death that resembles HR [47].

Forward genetics is one way to identify the genes that regulate preformed defense. Using QTL mapping, we show that preformed defense is amenable for genetics. Several regions of the rice genome controlling preformed expression of the *CHI *and *BURP *genes were identified (Figure 8). The architecture of this control is probably complex since our analysis of only two genes revealed six eQTLs. However, the observation that the *BURP *and *CHI *belong to the same regulon (Figure 4) is consistent with the observation that two eQTLs are common to these genes (Figure 8). These regions of chromosome 7 and 11 may contain regulators of preformed defense that are specific to the tropical japonica sub-groups. Fine mapping of these regions will help us identify the genes that control preformed defense and partial resistance.

More importantly, we show the first genetic evidence that two eQTLs controlling constitutive expression of the *CHI *and *BURP *genes co-localize with two QTLs for partial resistance. Given the number of genetic markers used for mapping (133), the number of QTL (11) and eQTL (6) found, the probability to find such co-localizations was very low (P = 0.011). A more detailed analysis will be necessary to establish a functional relationship between these two phenomena.

Interestingly, one of the eQTL controlling *CHI *constitutive expression co-localizes with the *CHI *structural gene on chromosome 7. Thus this eQTL could be a cis-eQTL. We did not find a potential cis-eQTL for the *BURP *gene, suggesting that constitutive expression for this gene is mostly controlled in *trans*. Given the yet imprecise position of the eQTLs, the eQTL controlling the *CHI *around the RG4 marker could also be a *trans *eQTL. In such a case, this region around RG4 marker on chromosome 7 could be a common regulator of constitutive expression for *BURP *and *CHI*.

Our QTL analysis already pinpoints some regulatory candidates that co-localize (4 Mb range) with several eQTLs. The eQTL on chromosome 1 co-localizes with *OsWRKY13 *[49]. This transcription factor has been shown to be involved in blast disease resistance as plants over-expressing *OsWRKY13 *show enhanced resistance to this pathogen. Plants over-expressing OsWRKY13 also displayed constitutive, elevated, levels of expression of defense-related genes but *PBZ1 *was down-regulated. Thus this gene is unlikely a good candidate for regulating preformed defense. The eQTL on chromosome 7 (close to the RG4 marker) co-localizes with the *OsDR8 *[41] and the *CIGR1 *[48] genes. Preliminary analysis of the *cigr1 *mutant suggests that this gene is not responsible for the eQTL (Blein M, XG and JBM, data not shown). The *OsDR8 *gene is involved in the vitamin B1 biosynthesis pathway and in thiamine accumulation [41] is also found within 4 Mb of the RG4 marker on chromosome 7. Interestingly, plants silenced for *OsDR8 *show increased susceptibility to *M. oryzae *and reduced accumulation, before infection (as well as after infection) of several *PR *genes, including *POX223 *but not *PBZ1*. This is only partly consistent with a possible role of *OsDR8 *in preformed of defense. Consistent with the implication of the thiamine pathway in preformed defense, thiamine is known to be an inducer of defenses in plants, including rice [44]. Finally, the eQTL close to marker RG351 on chromosome 7 co-localizes with the *rTGA2.1 *gene [55]. Although silencing of the *rTGA2.1 *gene increased the constitutive expression of defense-related genes, it is yet unknown whether this mutation affects resistance to *M. oryzae*. Such attempt to co-localize known regulatory genes with eQTL is overall risky and fine mapping will be required to identify the genes explaining these eQTLs.

### Preformed defense systems as a way to respond to environmental stresses in plants

Plants have evolved sophisticated inducible systems to respond to pathogen challenge [1]. Expression level polymorphism (ELP) has been shown to be important for the gene-for-gene resistance pathway [e.g. [30,31]] but there was up to now no indication that ELP could play a role in partial resistance. By looking at ELP in partial resistance of rice to *M. oryzae*, we provide several lines of evidence that constitutive expression of defense-related genes correlates with partial resistance in naturally occurring diversity. This is the first evidence of the role of constitutive expression of defense-related genes in disease resistance. Plants have deployed such a proactive strategy to face abiotic stresses [50,51]. For example, a large portion of the genes that are normally induced by zinc stress in *Arabidopsis thaliana *are constitutively highly expressed in *A. halleri*, a species of the *Arabidopsis *genus showing enhanced tolerance to zinc. Thus, constitutive expression of zinc-responsive genes has been proposed as a mechanism by which *A. halleri *naturally increases its tolerance to zinc [50]. Using a similar approach, Taji et al [51] showed that a large number of abiotic or biotic stress-inducible *Arabidopsis thaliana *genes were expressed under normal growth conditions in salt cress (*Thellungiella halophila*), a naturally salt tolerant plant specie. Thus plants seem to have evolved proactive, non-inducible systems to face abiotic stresses.

Therefore, it appears that constitutive expression of the adapted repertoire of genes is a general strategy used by plants to face environmental pressure. This is consistent with our current knowledge on trait evolution which poses that regulatory polymorphism might better account for phenotypical variability than structural polymorphism [52].

### Fitness benefits and costs of preformed defenses

Fitness costs can explain the evolution and maintenance of induced resistance in plants. In fact, it is generally believed that inducible defenses have evolved to save energy under enemy free conditions, but costs still arise upon activation of these defenses under hostile condition [58]. However, van Hulten et al [59] have shown that benefits of priming-mediated resistance outweigh its cost if the environment imposes relatively high levels of disease pressure. Thus preformed defense may not be so costly, assuming a high and constant pressure from the pathogen.

We find that preformed expression of defense-related genes in rice affects rice blast but not bacterial blight resistance. During evolution in rice, a constant infection pressure by *M. oryzae *must have driven the selection towards this phenotype. Indeed, rice and *M. oryzae *have been found associated for a very long time [60]. It is thus possible that during evolution, a constant infection pressure by *M. oryzae *must have driven the selection towards the maintenance of preformed defenses, because in this recurrent disease environment, preformed defenses benefits outweigh its costs.

## Conclusions

Past research has largely focused on inducible mechanisms to explain disease resistance. We provide three lines of evidence that constitutive expression of defense-related genes significantly contributes to partial resistance. The role of preformed defense is supported by our diversity analysis, our analysis of the phenomenon during development and genetic evidence. Besides the fundamental aspect of this finding, this work also has important consequences for the breeding strategies. Although indica and japonica sub-groups show some differences in their ability to express preformed defense, this study shows that constitutive expression of defense-related genes is a good prediction tool for identifying rice accessions with elevated partial resistance, a form of durable resistance. It remains to establish whether this phenomenon is observed in other plant species. We encourage colleagues to revisit their repertoire of inducible genes in the light of our finding.

## Methods

### Rice accessions

Rice diversity was estimated from Garris et al [53]. The names used for the rice accessions are the names used for the mini Germplasm Bank. Seeds were obtained from CIRAD-Center for Biological Resource (France). Rice was grown as in [11].

### Selection of marker genes for gene expression studies

Three types of genes along the disease resistance pathway were selected: one *PRR*, 12 regulators and 12 defense genes (Additional file 4). This classification of genes was sometimes arbitrary as for some genes the putative function was unknown (e.g. 33 kDA secretory protein). The role of some putative regulator genes in rice was deduced from gene expression studies (e.g. the *EDS5 *gene) [11] and by transcriptome information gathered in the Archipelago database [54,12]. The *NPR1 *[8], *RCI1 *[36] and *EIN2 *[35] genes were included as markers for the salicylic acid, the jasmonic acid and the ethylene pathways respectively. Other genes were included in this study as regulator genes (*HLHDB*, *ZnFg1*, *ZnFg2 *and ZnPgXS) given their annotations and expression studies (Additional file 4). Defense genes were genes for which the annotation and expression studies suggest a direct role in limiting pathogen growth. For example the *CHI *gene potentially degrades chitin, the major component of fungal cell-wall. Altogether, these genes are representative of the defense arsenal. All genes used in this work were, to some extent, differentially expressed upon infection (Additional file 4).

### Fungal quantification *in planta *and evaluation of partial resistance

Twenty-eight cultivars were characterized for partial resistance (Additional File 2). Plants were grown and inoculated when 3-weeks old with spore suspensions of 50000 spores/mL as in [11]. The quantity of fungal mass for four isolates of *M. oryzae *(CD101, CD203, CL26, CM28) was measured by Q-PCR on DNA extracted 7 days post-inoculation, in three independent experiments (8 leaves/experiment). Fungal growth was estimated using Taqman^® ^technology with the *MAGGY *transposon for *M. oryzae *(*MAGGY *Taqman probe TGAGCAGCCAACGCCGCCACAA) and the *ACTIN *gene for rice (*ACTIN *Taqman probe ATCACGCCCAGCAAGGTCGAGACG). Primers are given in Additional File 13. The Eurogentec Taqman kit was used on a Stratagene MX300P QPCR machine. In addition to classical symptoms (data not shown), a total of 12 values (4 isolates X 3 biological replicates) were used to build the partial resistance index. The inverse of the mean of the 12 measures obtained per cultivar was assumed to be an estimation of partial resistance (Additional File 2).

### Gene expression analysis

RNAs were extracted and gene expression measured as in [11]. All expression experiments were done two to three times in biologically independent experiments. The primers used are listed in Additional File 13. Calculation of gene expression was normalized using the rice *ACTIN *gene and expression formula from Pfaffl [56]. Although naturally occurring DNA polymorphism only slightly modifies gene expression using oligo-nucleotide microarrays [29], QRT-PCR that involves longer DNA sequences could be sensitive to DNA polymorphism. Thus we evaluated the variability of QRT-PCR measures for eight genes in six representative rice accessions (Additional File 14). Four genes (*33 kDA*, *BURP*, *CHI *and *HSP90*) showed QRT-PCR efficiencies more variable than in the *ACTIN *control but the overall variation was low (<20%). This variability was not related to indica/japonica sub-group or elevated/low partial resistance classes. The four other genes tested (*PBZ1*, *HLHDB*, *SPL7 *and *ZnFg2*) showed very limited variability across rice diversity. We concluded that the QRT-PCR conditions used in this study, although influenced by DNA polymorphism, were sufficient to evaluate expression variability across rice diversity.

### Statistical analysis of the data

The Pearson correlation coefficient value and the test of the value being different from zero were estimated with functions "cor" and "cor.test" in R Stats package (http://www.R-project.org). For Principal Component Analysis (PCA), the numeric variables were log2 transformed. PCA were done with function "dudi.pca" in ade4 library (http://pbil.univ-lyon1.fr/ADE-4) for R (http://www.R-project.org). For ANOVA, the variables were log2 transformed. The ANOVA models "*M. oryzae *quantity = gene 1 expression value + gene 2 expression value + ... + gene x expression value" were tested with the "lm" function in R Stats package. Models were validated with Shapiro.test function (residues normality) in R Stats package and hmctest or bptest functions (heteroskedasticity) in lmtest library (http://cran.r-project.org/web/packages/lmtest/index.html) for R. Models explanation was done with the anova function of R Stats package.

### Salicylic acid and ethylene quantification

For SA measurements, 3-week old plants were used. We only measured total SA accumulation as it mirrors free SA accumulation [61]. Frozen (liquid nitrogen) leaf tissues (about 0.5 g) were ground in 0.5 ml of 90% methanol and [14^C^] SA was then added (60 μl) as tracer to each tube. After centrifugation (15 min, 16000 g), the residue was extracted again with 100% methanol (0.5 ml) and, after centrifugation (15 min, 16000 g), the second supernatant was added to the first one. After a third centrifugation (10 min, 16000 g) combined supernatants were evaporated to dryness with a Speedvac (5 h, 30°C). For each sample, the dried extract was resuspended in hot water (80°C, 0.4 ml) and HCl 12 N (0.2 ml) and incubated for 45 min at 80°C in a water bath. After cooling, 1 ml of ether was added and after centrifugation, the organic phase was collected. A new step of phase partitioning was achieved on the aqueous phase. The two organic phases were then added and evaporated to dryness under nitrogen flux. Final samples were resuspended in 200 μl of injection buffer (10% acetonitrile, 90% sodium acetate 20 mM, pH 5.0) and 50 μl of a tenth dilution was used for injection.

Total SA was measured by fluorescence (λ_ex _313 nm, λ_em _405 nm) with a Nova-Pak 4-mm C-18 column (150 × 3.9 mm; Waters) as part of the Waters system (1525 Binary HPLC Pump, 2475 Multi λ Fluorescence Detector, 2996 Photodiode Array Detector, 717 Autosampler; Waters). Data (retention time and Area) were analyzed using Empower Pro Software (Waters). Radioactivity was determined by liquid scintillation counting of an aliquot sample. Recoveries of the internal standard [14^C^] SA were between 20 and 100% and for each sample, this yield was considered in SA quantity calculation. SA quantity was calculated as followed:

(quantity of SA (ng)/fresh weight (g)). = dilution factor × ([{(area/SA quantity standard curve slope) × (resuspending volume/injection volume)}/yield]/fresh weight (g)).

For ethylene measurements, plants were grown under sterile conditions and leaves were harvested and weighted after two weeks. Ethylene was extracted and measured as in [35].

### QTL and eQTL identification

The MapDisto free software (http://mapdisto.free.fr/) was used for QTL and eQTL analysis. Two mapping populations were used: a population of 60 RILs between Moroberekan and Co39 [38] and another between Azucena and IR64 with 84 RILs [39]. The gene expression and disease values were log2 transformed. The distributions of the resulting values followed a normal distribution (tested with Shapiro.test function in R Stats package; data not shown) and were used for QTL analysis.

## Authors' contributions

EV, XG and VC carried out the molecular, genetic and physiological studies.

PS designed the experiments and participated in the analysis of salicylic acid.

EB participated in the design of the study and performed part of the statistical analysis.

JBM, DT and JLN conceived the study and participated in its design and coordination.

All authors read and approved the final manuscript.

## Supplementary Material

Additional file 1 **Genetic diversity of rice cultivars used in this study**. Because seed stocks can sometimes degenerate, eleven microsatellites were used to confirm sub-group (japonica or indica) assignation of the rice cultivars used. Darwin (http://darwin.cirad.fr/darwin/Home.php) was used to build the dendrogram. The values represent the robustness based on 1000 bootstraps. Click here for file

Additional file 2 **List of cultivars characterized for their basal resistance to blast disease**. Twenty-eight cultivars were initially characterized, 13 Indica cultivars, eight tropical Japonica cultivars and seven temperate Japonica cultivars. The quantity of four isolates of *M. oryzae *(CD101, CD203, CL26, CM28) were measured by Q-PCR in planta 7 dpi in three biological repetitions. The darker the color is, the more the fungus is present. The inverse of the mean of the 12 measures obtained for each cultivar was used as an estimation of partial resistance. Measures lower than 1.00E-05 (black frame) were removed of the calculation because considered as measures of complete resistance. Click here for file

Additional file 3 **Partial resistance and constitutive defense expression indexes**. Origin of the cultivars selected for evaluation of basal resistance and gene expression studies. The partial resistance value is the mean of 12 measures of fungal growth using four different multivirulent isolates (see Methods and Additional File 2). The preformed-constitutive expression index was calculated according to Additional File 5 using 21 genes (Additional File 4). NO_GBI: IRGC number Click here for file

Additional file 4 Genes used in this study Click here for file

Additional file 5 **Index of gene expression level**. Example of calculation of gene expression index. Three steps were used for the calculation of the preformed defense index. 1 - For each gene, the mean is calculated for the 23 cultivars. 2 - the expression value for each gene in each cultivar is then divided by the mean expression level. 3 - for each cultivar, the mean for the 21 genes selected is calculated Click here for file

Additional file 6 **Correlation between partial resistance and constitutive or inducible expression of defense genes**. The log value of partial resistance index (Y-axis; Additional file 4) and expression of preformed expression of 21 genes index (X-axis; Additional File 3) of the six representative rice cultivars (Figure 3) was plotted for each time point before (A) and during infection (1 dpi: B, 2 dpi: C and 3 dpi:D). Correlation coefficients were statistically tested using the Pearsons' product moment correlation coefficent test. Click here for file

Additional file 7 **Constitutive expression of defense genes across rice diversity**. Gene expression was measured by QRT-PCR, normalized using actin and values are given in arbitrary unit (au). The vertical lines separate, from left to right, indica, temperate japonica and tropical japonica genotypes. The POX223 (**A**), RBBI2 (**B**), PBZ1 (**C**) and BURP (**D**) genes are shown for each cultivar (black bars). The mean of each genetic subgroup of cultivars is also indicated (grey bars). In each genetic subgroup, the genotypes are ranked from the less to the most resistant (according to Figure 1). Click here for file

Additional file 8 **Partial resistance and constitutive expression in different rice subgroups**. The log value of partial resistance (X-axis; Additional file 3) and expression of preformed expression of 21 genes (Y-axis; Additional File 4) indexes of the 12 indica (A) and 11 japonica (B) representative rice cultivars was plotted. Correlation coefficients were statistically tested using the Pearsons' product moment correlation coefficient test and the Bonferroni correction (the initial 0.01 threshold was divided by 3 because each data set was tested 3 times). Click here for file

Additional file 9 **Principal Component Analysis of preformed expression of defense**. A principal component analysis (PCA) was done using the expression values of 21 genes (Additional File 4) in 23 rice genotypes (Figure 1) for three independent experiments. The two axes represented of this PCA represent 43% and 56% of variability for indica and japonica respectively. For graphical purpose, the reverse value of partial resistance was plotted and designated by "*M. oryzae"*. Thus, genes that are located in the left part of the figure (e.g. *PBZ1*) have a constitutive expression that seems to correlate with partial resistance. A similar analysis was done for the indica (A) and the japonica (B) sub-groups of rice (Additional File 3) and used for the ANOVA analysis summarized in the Additional File 3. Click here for file

Additional file 10 **ANOVA analysis of preformed expression of defense**. a: The model of the ANOVA test was « *M. oryzae *quantity after inoculation = constitutive expression of gene 1 + constitutive expression of gene 2 +...constitutive expression of gene X + residual». b: correlation value between constitutive expression of each gene and basal resistance as estimated by PCA. When there was no apparent possible correlation in the PCA analysis (NL: no link; Additional File 9), the test was not done (nt: not tested). c: Early time points in the kinetic are 1 and 2 dpi, late time points are 3 and 4 dpi. +: Induction; -: repression; NC: no change in the expression. The CD203 isolate of *M. oryzae *was used. Click here for file

Additional file 11 **Partial resistance increases during plant development**. Gene expression was measured before infection on plants of different stages (2 to 8 weeks). The level of expression was measured in the before the last (n-1) and the last emerged (n) leaves. The ratio (n-1)/n was calculated and is shown for two genotypes: Moroberekan (A) and Azucena (B). Click here for file

Additional file 12 **LOD score and position of the QTL and eQTL**. Resistance (R) was evaluated as well as the expression, before infection, of the BURP and CHI genes (eQTL) using the Moroberekan X Co39 mapping population. The QTLs and eQTLs were detected using the MapDisto software. Two replicates were done; the LOD score is indicated for each position and character. Click here for file

Additional file 13 Primers used in this study Click here for file

Additional file 14 QRT-PCR amplification efficiency of selected primer pairsacross rice diversity Click here for file
