# Supplementary figures and images for: Preformed expression of defense is a hallmark of partial resistance to rice blast fungal pathogen Magnaporthe oryzae
Source: BMC Plant Biol. 2010 Sep 17;10:206. doi: 10.1186/1471-2229-10-206 (PMC2956555; doi:10.1186/1471-2229-10-206)

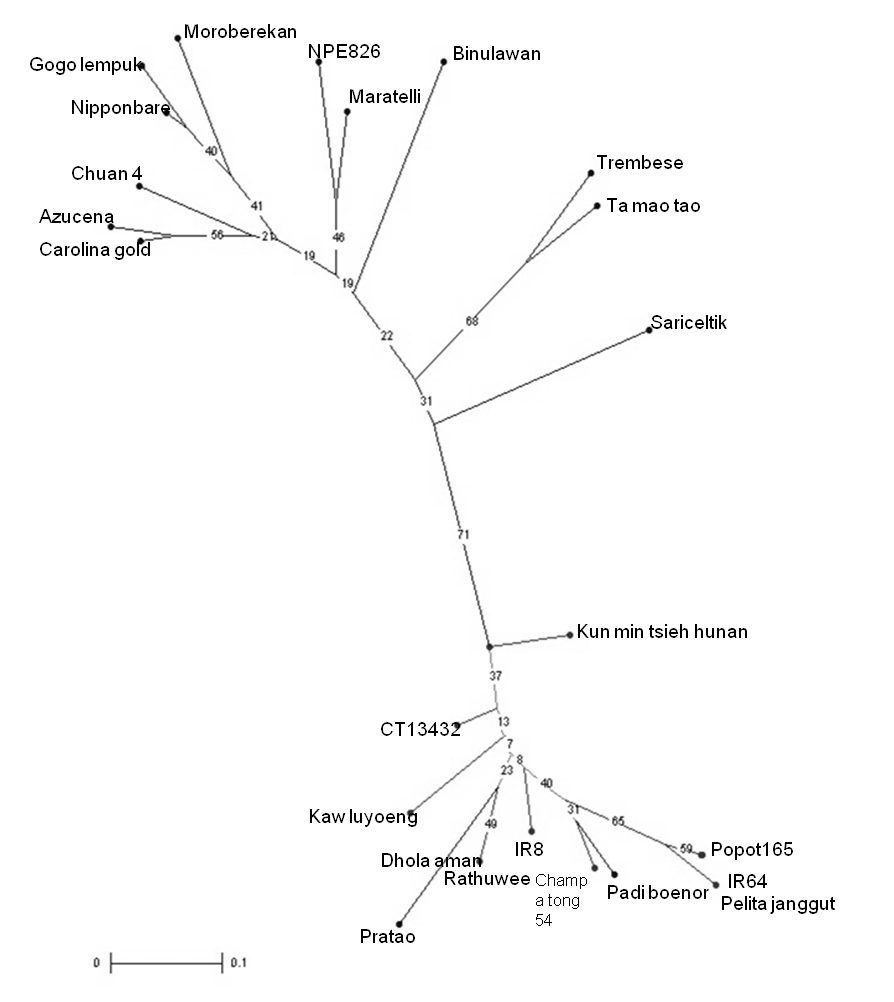

Supplement: Additional file 1 — Genetic diversity of rice cultivars used in this study. Because seed stocks can sometimes degenerate, eleven microsatellites were used to confirm sub-group (japonica or indica) assignation of the rice cultivars used. Darwin (http://darwin.cirad.fr/darwin/Home.php) was used to build the dendrogram. The values represent the robustness based on 1000 bootstraps. [file 1471-2229-10-206-S1.PNG]

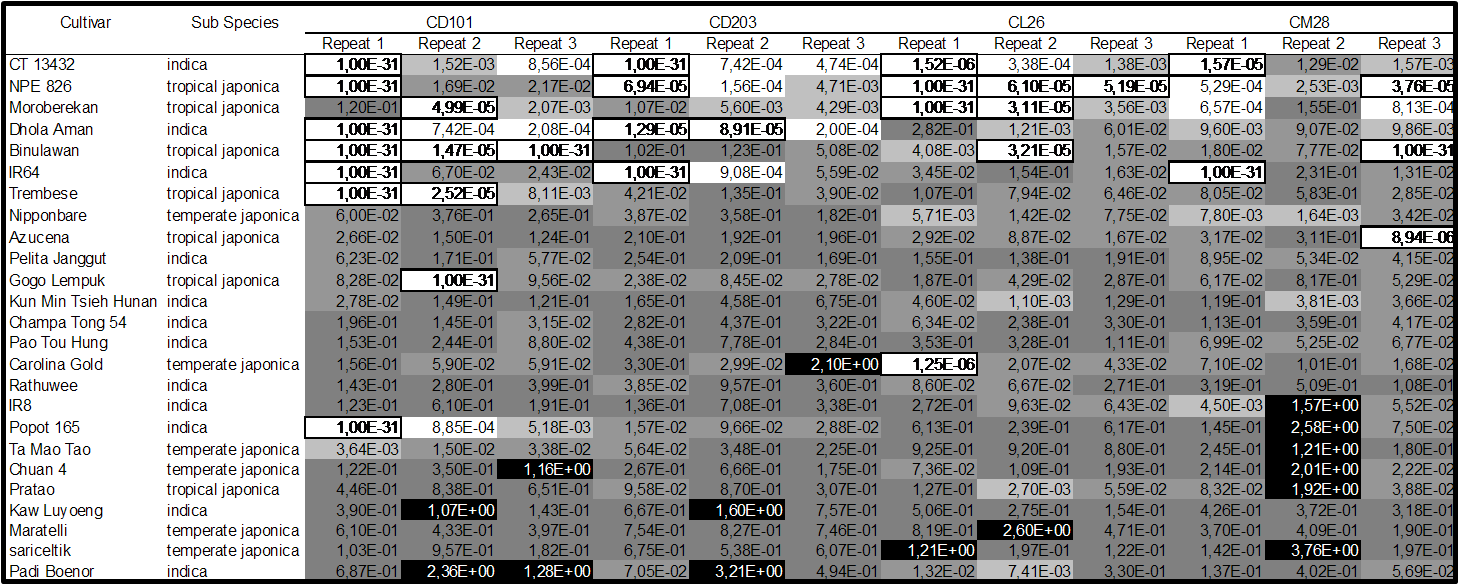

Supplement: Additional file 2 — List of cultivars characterized for their basal resistance to blast disease. Twenty-eight cultivars were initially characterized, 13 Indica cultivars, eight tropical Japonica cultivars and seven temperate Japonica cultivars. The quantity of four isolates of M. oryzae (CD101, CD203, CL26, CM28) were measured by Q-PCR in planta 7 dpi in three biological repetitions. The darker the color is, the more the fungus is present. The inverse of the mean of the 12 measures obtained for each cultivar was used as an estimation of partial resistance. Measures lower than 1.00E-05 (black frame) were removed of the calculation because considered as measures of complete resistance. [file 1471-2229-10-206-S2.PNG]

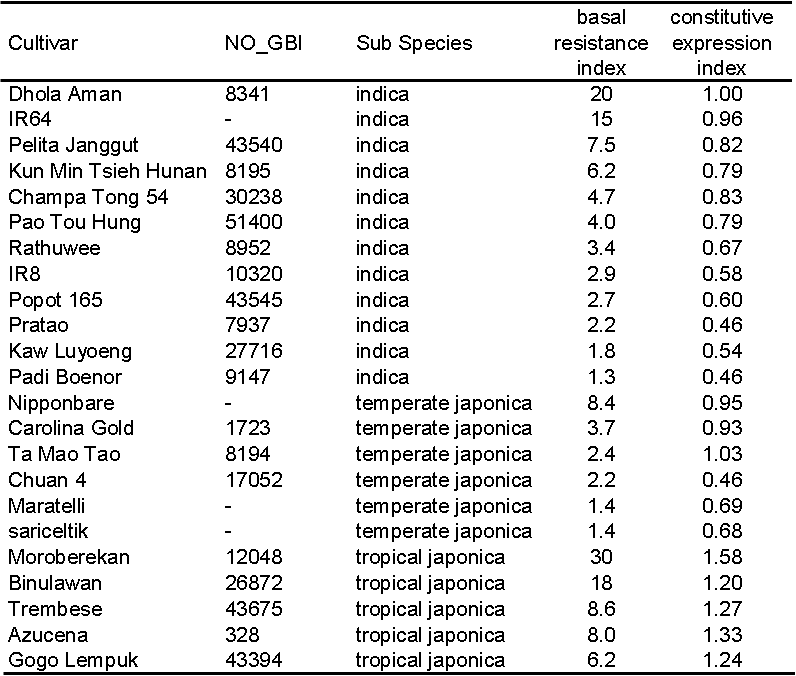

Supplement: Additional file 3 — Partial resistance and constitutive defense expression indexes. Origin of the cultivars selected for evaluation of basal resistance and gene expression studies. The partial resistance value is the mean of 12 measures of fungal growth using four different multivirulent isolates (see Methods and Additional File 2). The preformed-constitutive expression index was calculated according to Additional File 5 using 21 genes (Additional File 4). NO_GBI: IRGC number [file 1471-2229-10-206-S3.PNG]

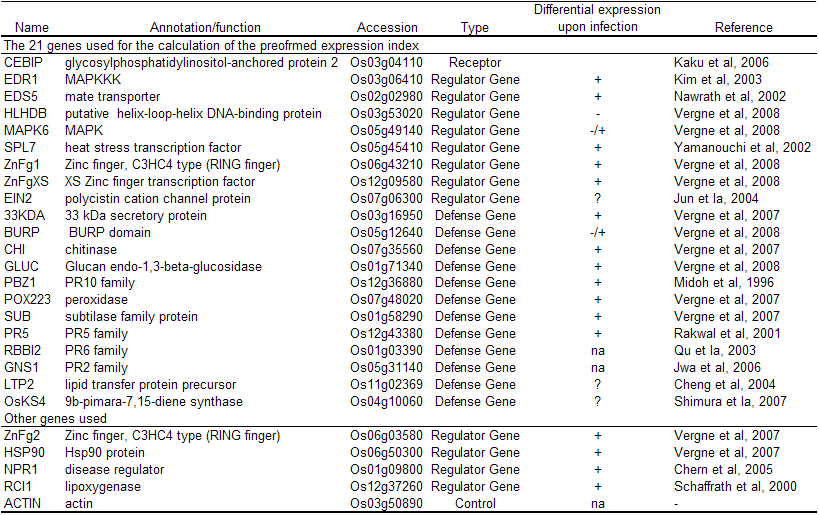

Supplement: Additional file 4 — Genes used in this study [file 1471-2229-10-206-S4.PNG]

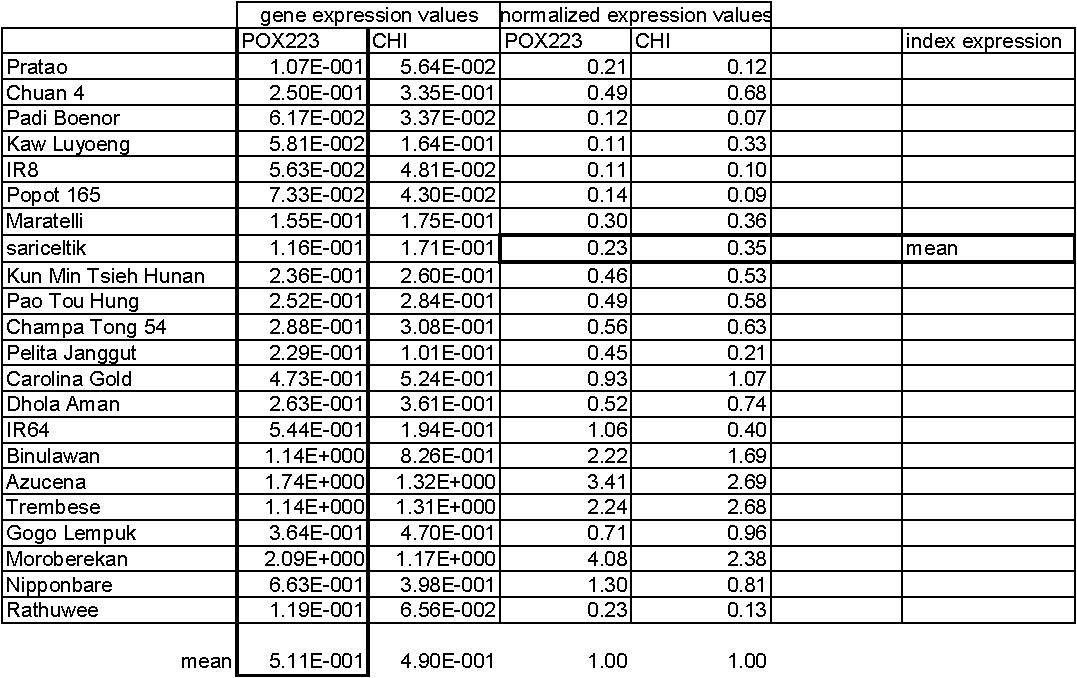

Supplement: Additional file 5 — Index of gene expression level. Example of calculation of gene expression index. Three steps were used for the calculation of the preformed defense index. 1 - For each gene, the mean is calculated for the 23 cultivars. 2 - the expression value for each gene in each cultivar is then divided by the mean expression level. 3 - for each cultivar, the mean for the 21 genes selected is calculated [file 1471-2229-10-206-S5.PNG]

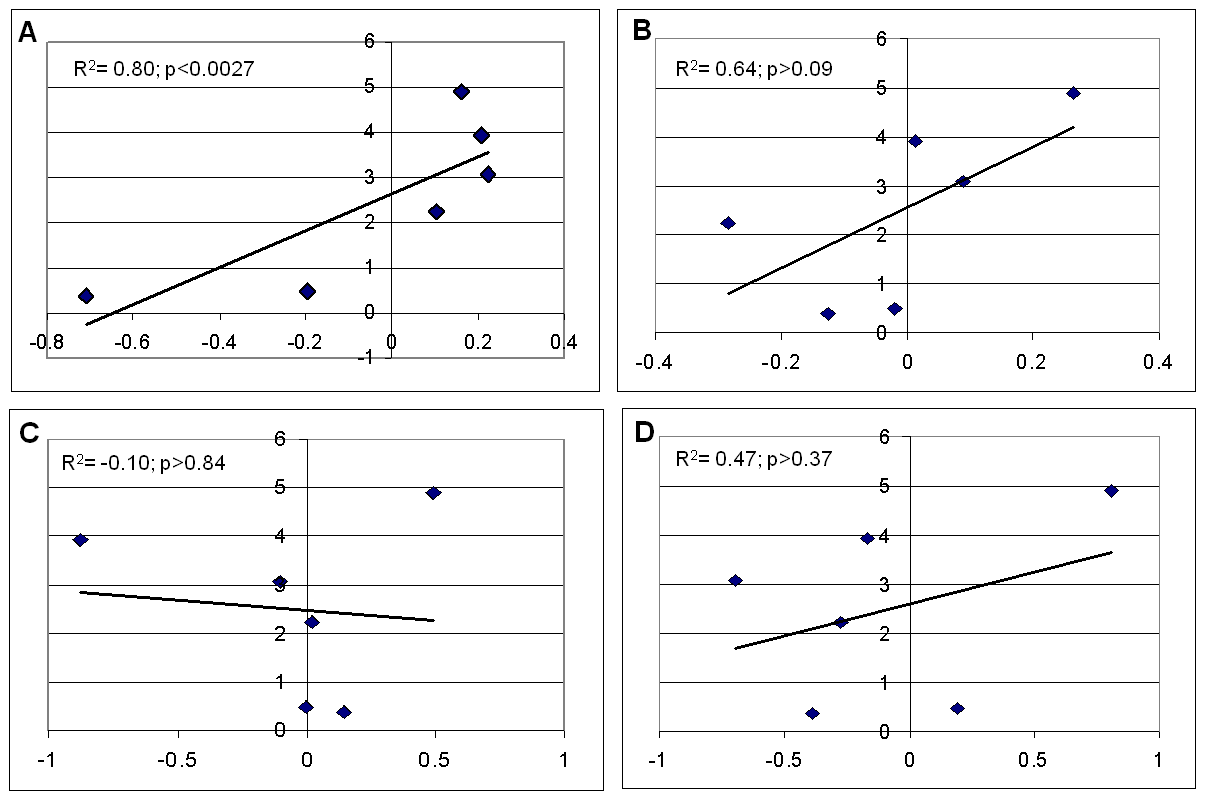

Supplement: Additional file 6 — Correlation between partial resistance and constitutive or inducible expression of defense genes. The log value of partial resistance index (Y-axis; Additional file 4) and expression of preformed expression of 21 genes index (X-axis; Additional File 3) of the six representative rice cultivars (Figure 3) was plotted for each time point before (A) and during infection (1 dpi: B, 2 dpi: C and 3 dpi:D). Correlation coefficients were statistically tested using the Pearsons' product moment correlation coefficent test. [file 1471-2229-10-206-S6.PNG]

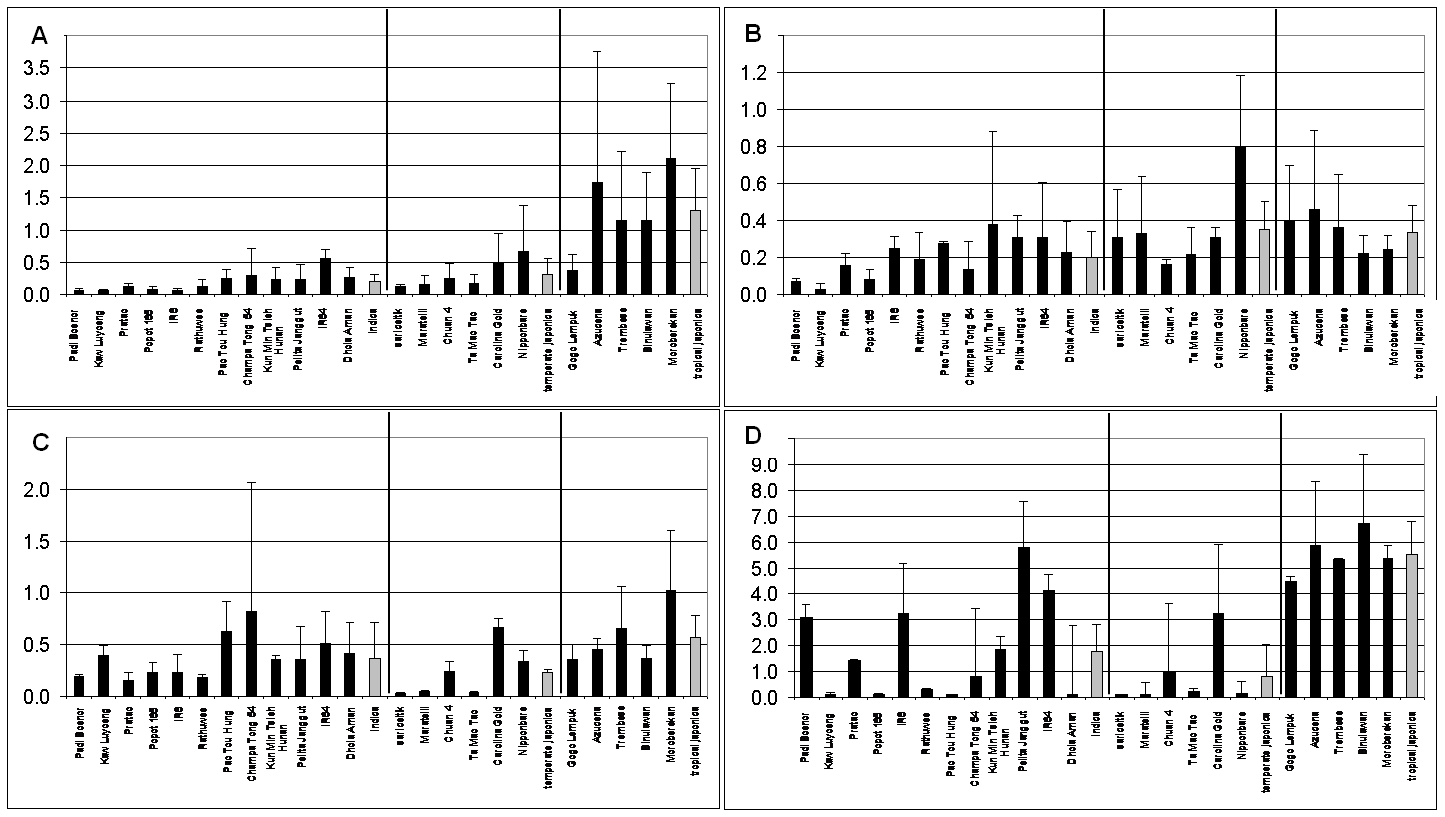

Supplement: Additional file 7 — Constitutive expression of defense genes across rice diversity. Gene expression was measured by QRT-PCR, normalized using actin and values are given in arbitrary unit (au). The vertical lines separate, from left to right, indica, temperate japonica and tropical japonica genotypes. The POX223 (A), RBBI2 (B), PBZ1 (C) and BURP (D) genes are shown for each cultivar (black bars). The mean of each genetic subgroup of cultivars is also indicated (grey bars). In each genetic subgroup, the genotypes are ranked from the less to the most resistant (according to Figure 1). [file 1471-2229-10-206-S7.PNG]

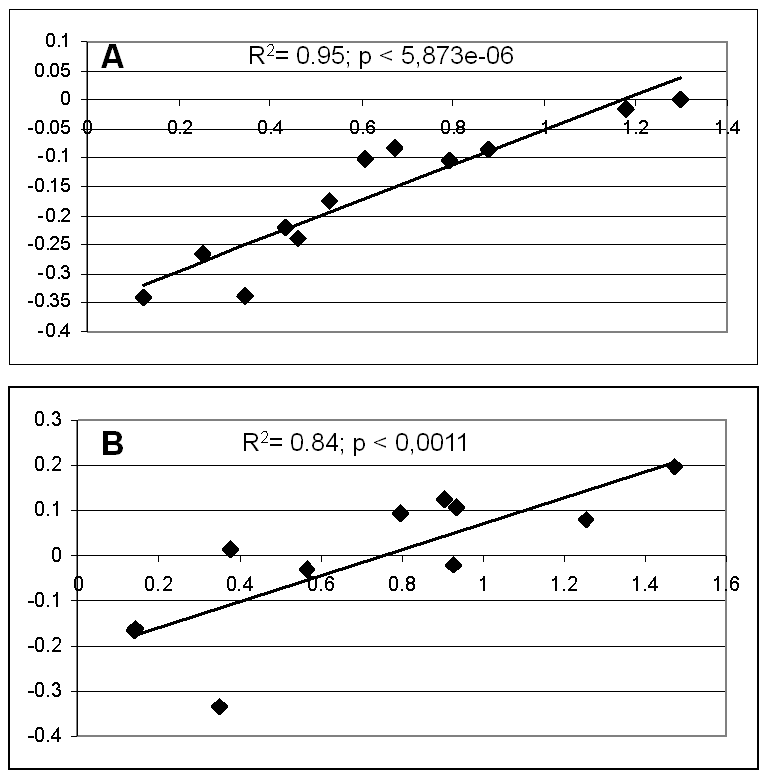

Supplement: Additional file 8 — Partial resistance and constitutive expression in different rice subgroups. The log value of partial resistance (X-axis; Additional file 3) and expression of preformed expression of 21 genes (Y-axis; Additional File 4) indexes of the 12 indica (A) and 11 japonica (B) representative rice cultivars was plotted. Correlation coefficients were statistically tested using the Pearsons' product moment correlation coefficient test and the Bonferroni correction (the initial 0.01 threshold was divided by 3 because each data set was tested 3 times). [file 1471-2229-10-206-S8.PNG]

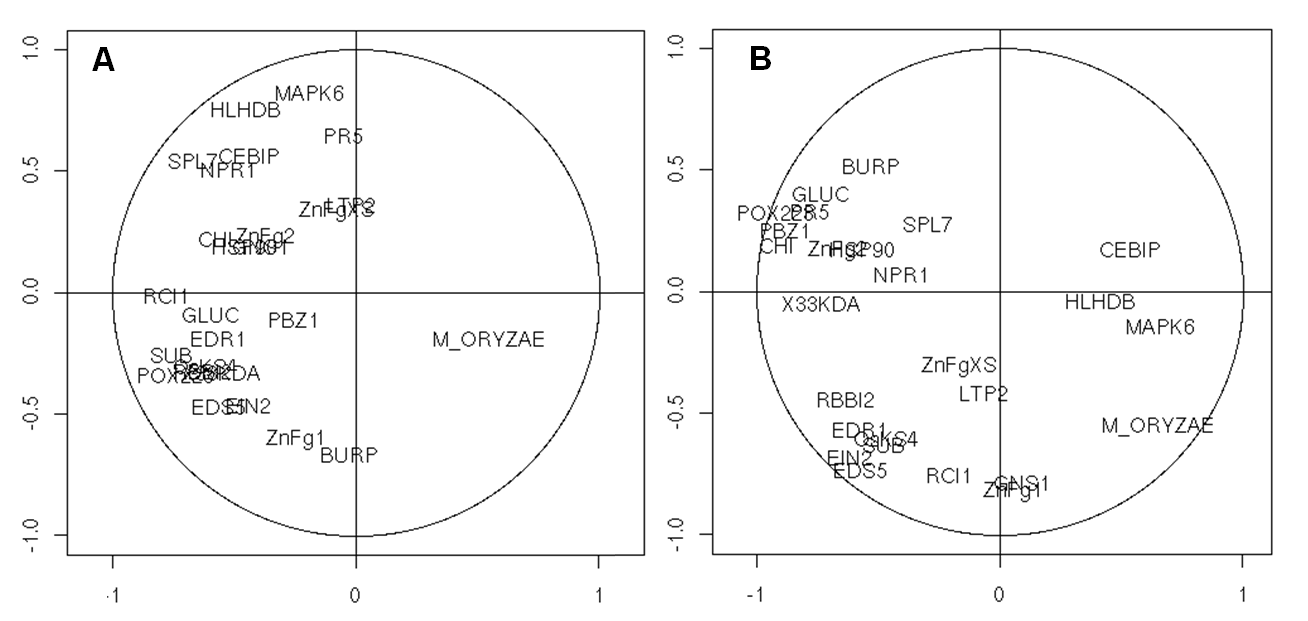

Supplement: Additional file 9 — Principal Component Analysis of preformed expression of defense. A principal component analysis (PCA) was done using the expression values of 21 genes (Additional File 4) in 23 rice genotypes (Figure 1) for three independent experiments. The two axes represented of this PCA represent 43% and 56% of variability for indica and japonica respectively. For graphical purpose, the reverse value of partial resistance was plotted and designated by "M. oryzae". Thus, genes that are located in the left part of the figure (e.g. PBZ1) have a constitutive expression that seems to correlate with partial resistance. A similar analysis was done for the indica (A) and the japonica (B) sub-groups of rice (Additional File 3) and used for the ANOVA analysis summarized in the Additional File 3. [file 1471-2229-10-206-S9.PNG]

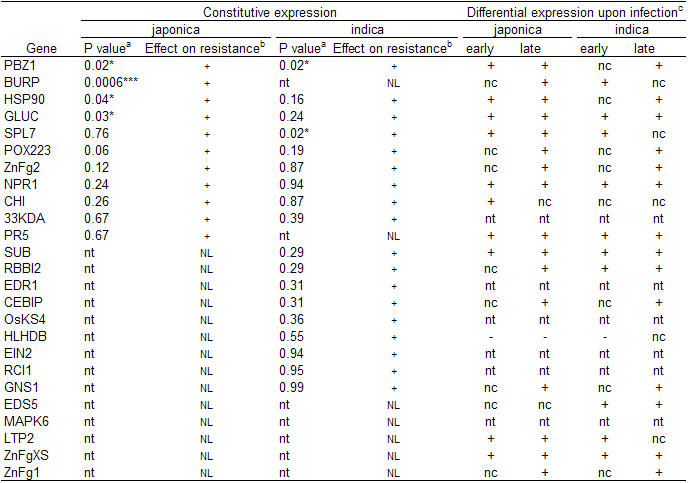

Supplement: Additional file 10 — ANOVA analysis of preformed expression of defense. a: The model of the ANOVA test was « M. oryzae quantity after inoculation = constitutive expression of gene 1 + constitutive expression of gene 2 +...constitutive expression of gene X + residual». b: correlation value between constitutive expression of each gene and basal resistance as estimated by PCA. When there was no apparent possible correlation in the PCA analysis (NL: no link; Additional File 9), the test was not done (nt: not tested). c: Early time points in the kinetic are 1 and 2 dpi, late time points are 3 and 4 dpi. +: Induction; -: repression; NC: no change in the expression. The CD203 isolate of M. oryzae was used. [file 1471-2229-10-206-S10.PNG]

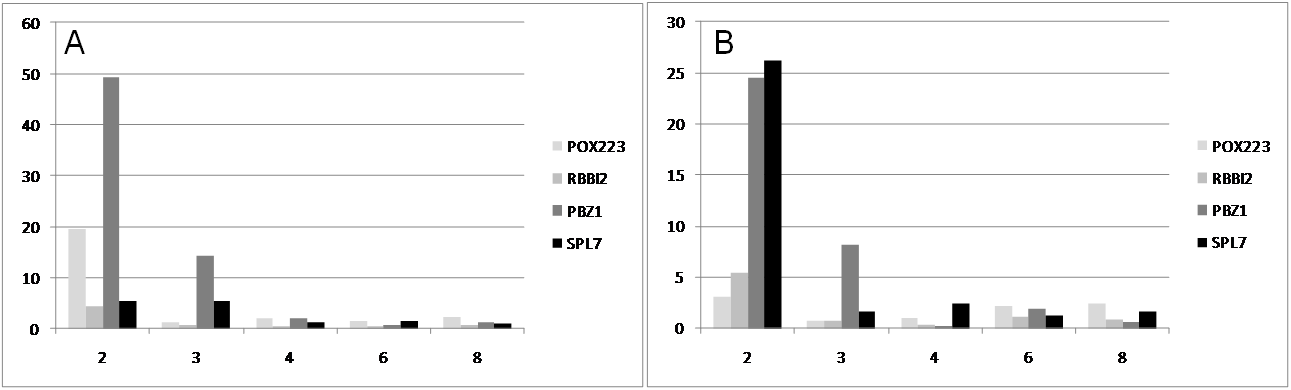

Supplement: Additional file 11 — Partial resistance increases during plant development. Gene expression was measured before infection on plants of different stages (2 to 8 weeks). The level of expression was measured in the before the last (n-1) and the last emerged (n) leaves. The ratio (n-1)/n was calculated and is shown for two genotypes: Moroberekan (A) and Azucena (B). [file 1471-2229-10-206-S11.PNG]

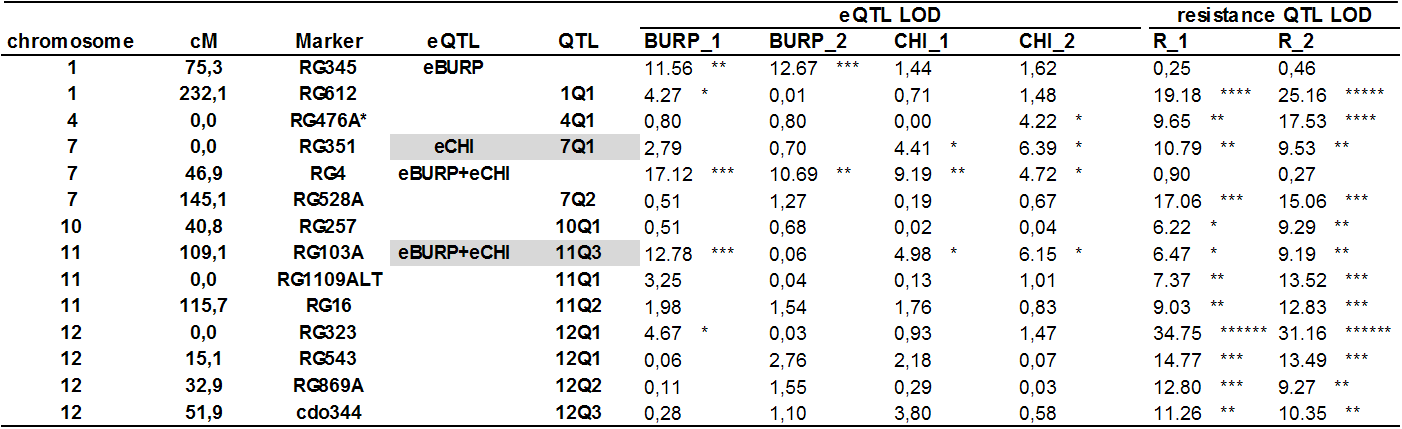

Supplement: Additional file 12 — LOD score and position of the QTL and eQTL. Resistance (R) was evaluated as well as the expression, before infection, of the BURP and CHI genes (eQTL) using the Moroberekan X Co39 mapping population. The QTLs and eQTLs were detected using the MapDisto software. Two replicates were done; the LOD score is indicated for each position and character. [file 1471-2229-10-206-S12.PNG]

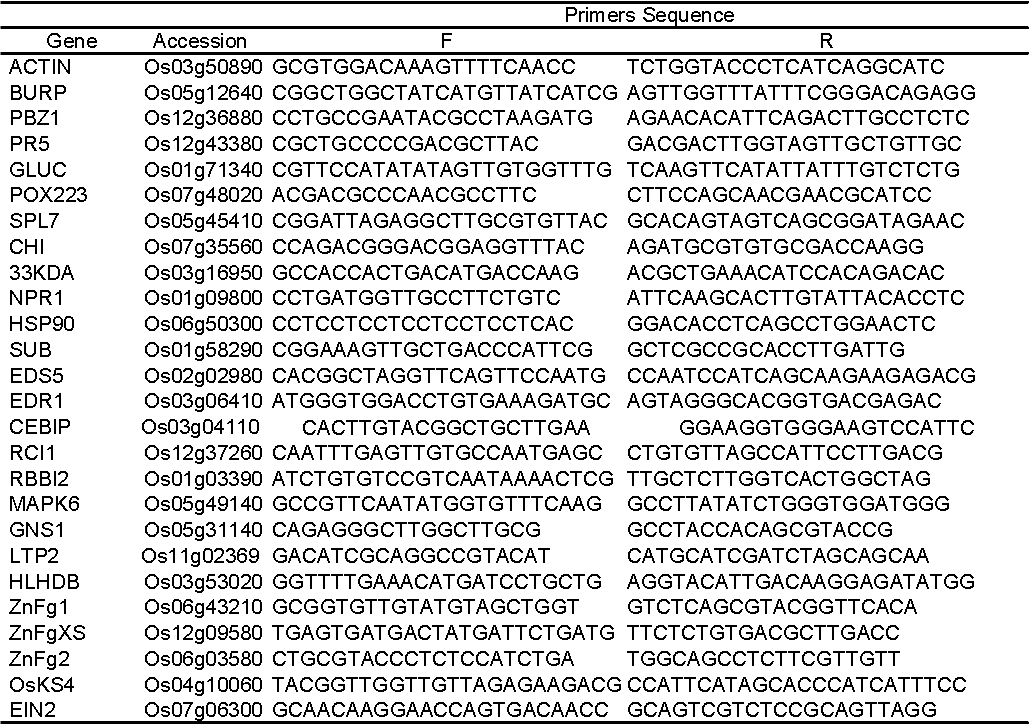

Supplement: Additional file 13 — Primers used in this study [file 1471-2229-10-206-S13.PNG]

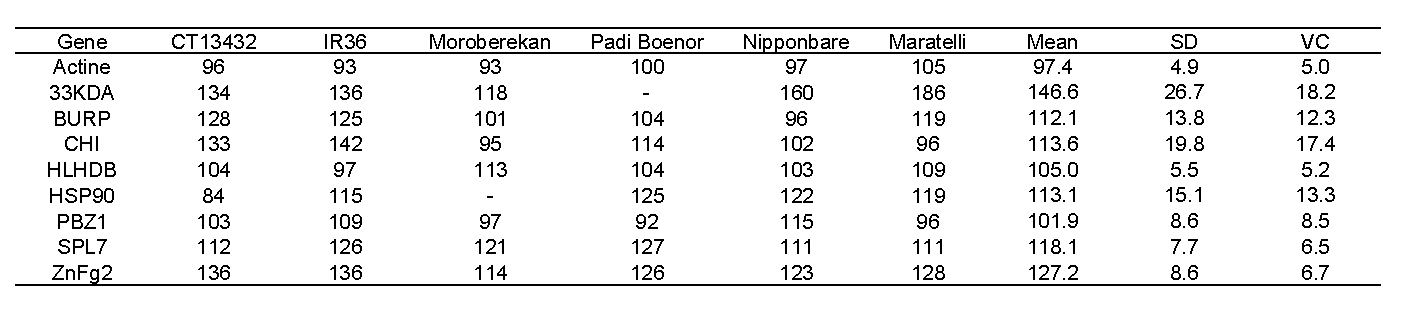

Supplement: Additional file 14 — QRT-PCR amplification efficiency of selected primer pairsacross rice diversity [file 1471-2229-10-206-S14.PNG]
